# Destructive Mold Osteomyelitis of the Wrist Caused by *Scedosporium apiospermum*—A Case Report

**DOI:** 10.3390/jcm15083035

**Published:** 2026-04-16

**Authors:** Camilla Bo, Anna Conen, Martina Giacalone, Regula Marti, Rainer Grobholz, Harald Seeger, Holger J. Klein, Jan A. Plock, Florian S. Frueh

**Affiliations:** 1 Department of Plastic Surgery and Hand Surgery, Kantonsspital Aarau, 5001 Aarau, Switzerland; camilla.bo1993@gmail.com (C.B.); martina.giacalone@students.uniroma2.eu (M.G.); holger.klein@ksa.ch (H.J.K.); jan.plock@ksa.ch (J.A.P.); 2 Clinic for Infectious Diseases and Infection Prevention, Kantonsspital Aarau, 5001 Aarau, Switzerland; anna.conen@ksa.ch; 3 Plastic and Reconstructive Surgery, Department of Surgical Sciences, Tor Vergata University of Rome, 00133 Rome, Italy; 4 Department of Vascular Surgery, Kantonsspital Aarau, 5001 Aarau, Switzerland; regula.marti@ksa.ch; 5 Medical Faculty, University of Zurich, 8032 Zurich, Switzerland; rainer.grobholz@ksa.ch; 6 Institute of Pathology, Kantonsspital Aarau, 5001 Aarau, Switzerland; 7 Department of Nephrology, Kantonsspital Baden, 5404 Baden, Switzerland; harald.seeger@ksb.ch

**Keywords:** free tissue flaps, hand surgery, immunocompromised host, osteomyelitis, *Scedosporium*, mycoses, wrist joint

## Abstract

**Background**: Wrist osteomyelitis caused by *Scedosporium apiospermum* is exceedingly rare. Its indolent course and destructive potential may result in extensive bone loss and pose substantial diagnostic and therapeutic challenges. **Methods**: We report a case of chronic wrist osteomyelitis caused by *Scedosporium apiospermum* in a 68-year-old kidney–pancreas transplant recipient. **Results**: Following diagnosis, systemic antifungal therapy with voriconazole was initiated, and multiple surgical debridements were performed to achieve local disease control, resulting in a large defect of the carpus and distal forearm. Hand salvage was attempted using an osteocutaneous triple-barrel fibula flap. The postoperative course was complicated by congestion of the fibula skin island, which was managed with leech therapy. Subsequent infection with a multi-resistant *Aeromonas* spp. and *Morganella morganii* led to flap necrosis, ultimately requiring transradial forearm amputation. **Conclusions**: Destructive *Scedosporium apiospermum* osteomyelitis in immunocompromised patients is a major challenge for reconstructive surgeons. Interdisciplinary management is essential as mold eradication is only achievable through a combined surgical and antimicrobial approach. In advanced destructive osteomyelitis, the choice between limb salvage and amputation should be individualized, considering patient comorbidities, reconstructive risk, and patients’ preferences. This case highlights the importance of balancing careful indication and patient counseling in complex clinical scenarios.

## 1. Introduction

Fungal osteomyelitis is a rare but clinically relevant condition, as it is associated with significant morbidity, particularly in immunocompromised patients. Among mold infections, *Aspergillus* spp. represents the most common etiological agents, followed by *Mucorales* spp. The vertebral spine is the most frequently affected site, followed by the extremities and skull [1,2]. In recent years, an increasing incidence of fungal osteomyelitis has been reported, likely reflecting the growing prevalence of immunocompromised individuals [3].

The genus *Scedosporium* (*S*) comprises two medically relevant species: *S. apiospermum* and *S. prolificans.* These are ubiquitous filamentous fungi and *S. apiospermum* is found worldwide in temperate areas, mostly in soil, sewage, waste, vegetation, and polluted water [4,5]. The infection is acquired exogenously, either through spores inhalation, or by direct inoculation of fungal elements, such as in the setting of open fractures or traumatic wounds [6].

While immunocompetent patients usually present with localized cutaneous or subcutaneous infections, immunocompromised hosts are more susceptible to develop disseminated disease, frequently involving the lungs and central nervous system [7,8]. Diagnostic evaluation relies on radiological imaging, followed by histopathological detection of fungal elements in tissue biopsies and confirmation through fungal cultures or molecular diagnostic techniques [6,7,8,9]. Early diagnosis is essential to avoid delays in treatment, which generally requires antifungal therapy and surgical management, and therefore to increase survival rates [10]. The literature on upper-extremity infections caused by *S. apiospermum* is limited. Reported cases primarily involve tenosynovitis, and only a single case of wrist infection in an immunocompetent patient has been described [11,12].

We report a case of chronic wrist osteomyelitis caused by *S. apiospermum* in an immunocompromised male patient, highlighting the diagnostic and therapeutic challenges associated with this uncommon entity.

## 2. Case Report

A 68-year-old patient, a retired individual with an active lifestyle and left-hand dominance, presented to a regional referral hospital with a prolonged history of pain and swelling of the left wrist. His medical history included type 1 diabetes mellitus, and a combined kidney–pancreas transplant performed eleven years earlier, for which he was receiving immunosuppressive therapy with belatacept, mycophenolic acid and low-dose prednisone. Symptoms started three months earlier with progressive wrist swelling and pain, leading to a markedly reduced functional use of the affected limb and quality of life. Following referral, multiple aspirations of the swelling were performed without successful fluid collection, and repeated joint aspirations failed to detect microorganisms. No surgical biopsies were performed in the first six months after onset of symptoms. Magnetic resonance imaging (MRI) revealed extensive wrist arthritis with carpal involvement.

Based on these findings, the patient had undergone a 10-day hospitalization for suspected rheumatoid arthritis. Occupational therapy had been attempted under regional anesthesia.

**Figure 1:**
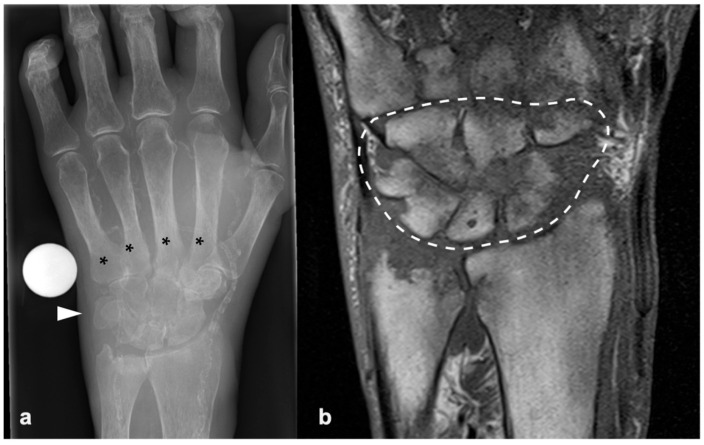
Preoperative imaging. (**a**) X-ray of the wrist with extensive bone destruction, affecting the whole carpus (arrowhead) and metacarpal bases (asterisks). (**b**) Magnetic resonance imaging of the wrist, highlighting arthritis and carpal bone osteomyelitis (dotted line).

Following progressive worsening of his symptoms, the patient was referred to our hand surgery center. At presentation, wrist mobility was severely limited due to pain. Further assessment with MRI showed extensive carpal destruction (Figure 1) and a proximal row carpectomy with histological and microbiological work-up was performed.

Histopathological examination confirmed chronic destructive osteomyelitis, with the presence of septate hyaline fungal hyphae within the bone tissue, consistent with hyalohyphomycosis. Microbiological cultures from bone tissue grew *S. apiospermum*. Species identification was established by a combination of methods: Colony morphology, microscopic examination of fungal elements, and Sanger sequencing of the ITS region using primers ITS1 (5′-TCCGTAGGTGAACCTGCGG-3′) and ITS4 (5′-TCCTCCGCTTATTGATATG-3′) following Clinical & Laboratory Standards Institute (CLSI) guidelines. Antifungal susceptibility testing with determination of minimum inhibitory concentrations (MIC) was done using E-test strips for amphotericin B, posaconazole, and voriconazole (bioMérieux, Marcy-l’Étoile, France), as well as for isavuconazole (Liofilchem, Roseto degli Abruzzi, Italy). MIC values were interpreted according to European Committee on Antimicrobial Susceptibility Testing (EUCAST) and CLSI guidelines (Table 1).

Table 1 Antimicrobial susceptibility testing. MICs were determined using E-test strips and interpreted according to EUCAST and CLSI guidelines. Since no species-specific breakpoints exist for *S. apiospermum*, MICs were referenced to breakpoints established for other filamentous fungi. CLSI: Clinical & Laboratory Standards Institute, EUCAST: European Committee on Antimicrobial Susceptibility Testing, MIC: Minimum inhibitory concentration.

No additional infectious foci were identified, and it was hypothesized that fungal inoculation might have occurred during gardening activities, although the patient denied any history of skin lesions. Voriconazole therapy was initiated with an intravenous loading dose of 400 mg twice daily on day 1 (2 × 6 mg/kg) and subsequently continued at 300 mg twice daily orally (2 × 4 mg/kg). Dose adjustments were performed as needed to achieve a target trough concentration of 2–6 mg/L, monitored by initially weekly therapeutic drug monitoring. During the first month of treatment, trough levels were 4.99 mg/L, 6.51 mg/L, 3.08 mg/L, and 2.43 mg/L, respectively. No adverse effects requiring interruption of therapy were observed. Adjunct systemic therapy with oral terbinafine 250 mg twice daily was initiated but discontinued after eight weeks because of poor tolerance (severe dizziness resulting in a fall) [9]. Thereafter, antifungal treatment was continued as voriconazole monotherapy, particularly in light of consistently negative follow-up fungal cultures. Local antifungal treatment with a polymethylmethacrylate cement spacer loaded with 0.4 g voriconazole per 40 g cement was added starting from the fourth debridement, when fungal infection was definitively diagnosed. The spacer was then replaced at each subsequent debridement. This approach aimed to optimize antifungal efficacy by combining systemic therapy with high-dose local delivery. Due to the severity of the infection, immunosuppressive treatment was reduced to the lowest feasible level. Mycophenolic acid dose was lowered (1080 to 720 mg daily), prednisone reduced (7.5 to 5 mg daily), and the belatacept interval extended (4-weekly to 5–6-weekly).

**Figure 2:**
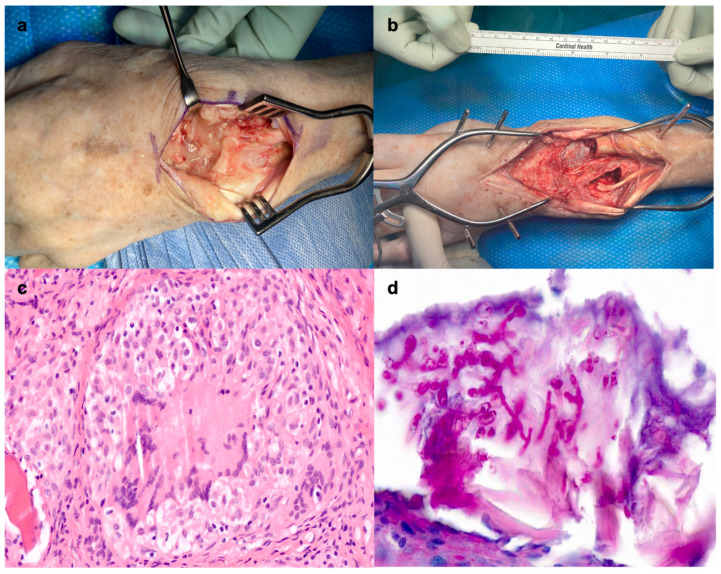
Intraoperative situs. (**a**) Intraoperative finding during the first debridement with pus in the radiocarpal joint. (**b**) Intraoperative finding after final bone resection with 11 cm bone defect of the wrist. (**c**) Histology showing osteomyelitis with granulomas and giant cells (Hematoxylin–Eosin staining, 400×). (**d**) Septate hyaline branching fungal hyphae consistent with hyalohyphomycosis; species identification was established by culture and molecular methods (PAS staining, 600×).

Subsequently, six further surgical debridements were necessary to obtain histological margins free of fungal elements. This ultimately resulted in extensive bone resection with an 11 cm defect and the need for external fixation (Figure 2). Follow-up fungal cultures were consistently negative.

**Figure 3:**
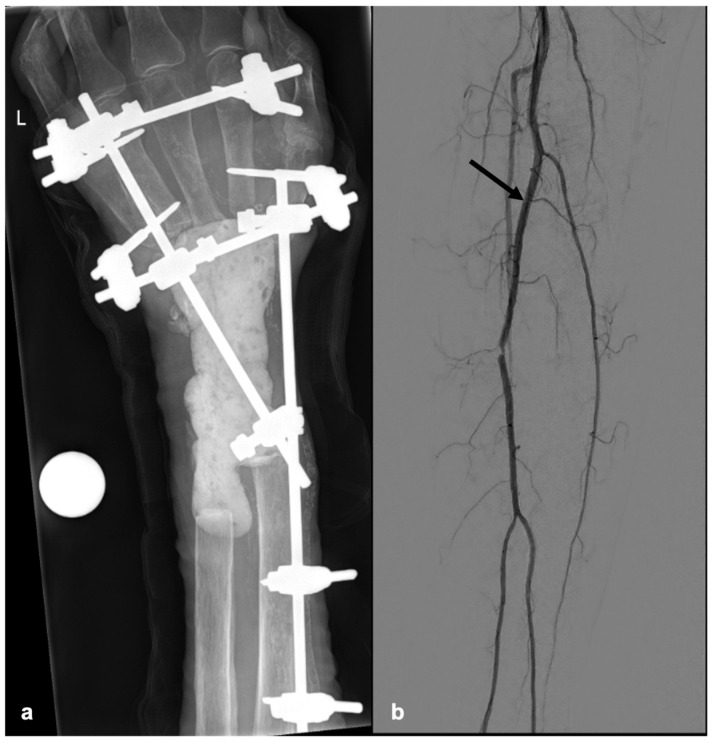
Preoperative planning. (**a**) X-ray of the wrist with extensive bone defect and local cement spacer and external fixation. (**b**) Lower-extremity arteriography with a peronea magna artery (black arrow) and severe arteriosclerosis.

Treatment options, i.e., reconstruction versus amputation, were discussed extensively with the patient. From a medical point of view, a transradial forearm amputation was recommended. Of note, the patient’s clinical situation was complicated by severe upper- and lower-extremity arteriosclerosis as well as an anatomical variant on the right lower leg with a peronea magna artery supplying the foot (Figure 3). The patient received psychological and psychotherapeutic counselling during hospitalization and on an outpatient basis prior to planning the reconstructive intervention. Despite these considerations, the patient repeatedly declined amputation.

**Figure 4:**
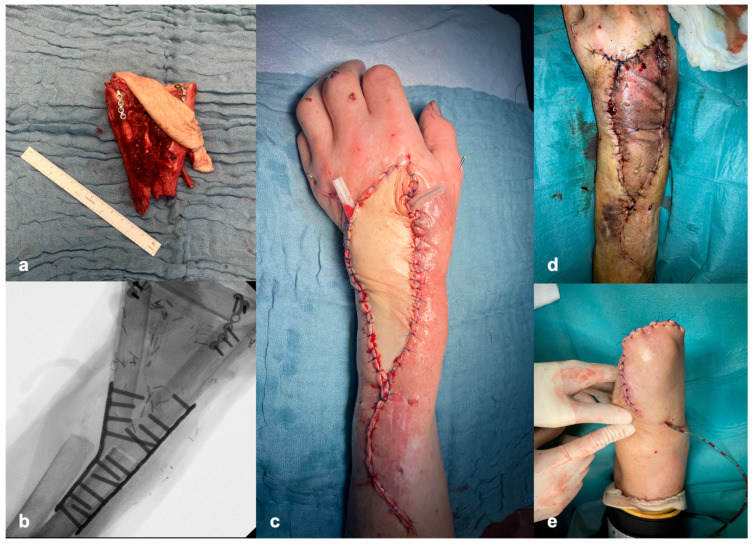
Reconstructive surgery. (**a**) Free osteocutaneous triple-barrel fibula flap. (**b**) X-ray of the wrist with radio-metacarpal reconstruction. (**c**) Clinical result after inset of flap. (**d**) Necrotic skin island eight days after reconstructive surgery. (**e**) Transradial forearm amputation.

After detailed informed consent, we performed a radio-metacarpal reconstruction using a microvascular osteocutaneous triple-barrel fibula flap (Figure 4). During the same operation, the foot was revascularized with a reverse basilic vein graft. Four days following reconstruction, the skin island of the fibula flap became congested, and was managed with medical leeching. Despite standard antibiotic prophylaxis with ciprofloxacin, leech therapy resulted in a severe bacterial soft tissue infection caused by two ciprofloxacin-resistant *Aeromonas* spp. (*A. hydrophila* and *A. veronii*), one of which was extended-spectrum beta-lactamase-producing, and *Morganella morganii* (Table 2), with subsequent necrosis of the flap.

**Figure 5:**
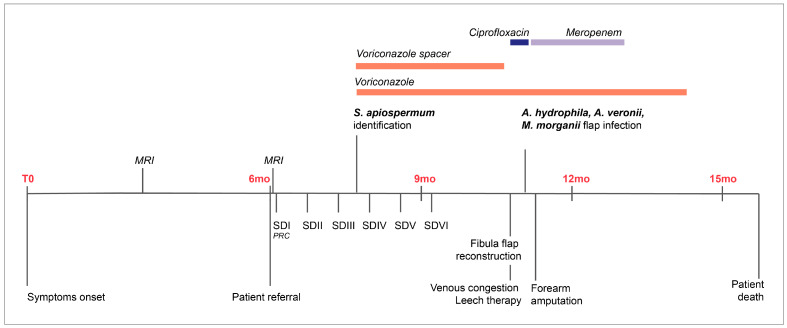
Chronological overview of the patient’s clinical course, including symptom onset, diagnostic workup, microbiological findings, and surgical interventions. MRI: magnetic resonance imaging, SD: surgical debridement, PRC: proximal row carpectomy.

Ultimately, a transradial forearm amputation was performed nine days after reconstruction, followed by uneventful wound healing under antimicrobial treatment with meropenem. Antifungal treatment was discontinued three months after the final surgery. The total duration of systemic voriconazole therapy was eight months. Two months later, the patient died due to cancer-related complications of a metastasizing skin cancer. The overall clinical course is summarized in Figure 5.

## 3. Discussion

Fungal wrist osteomyelitis is exceedingly rare, with only a limited number of cases reported in the literature [13]. We herein report a case of chronic destructive wrist osteomyelitis caused by *S. apiospermum* in an immunocompromised patient.

Infections of the upper extremity caused by *S. apiospermum* have been described in the literature, most commonly presenting as soft tissue infections or tenosynovitis [13]. Cases with osteoarticular involvement have also been reported, although they remain rare and are more frequently described in anatomical sites other than the wrist. Overall, confirmed cases of destructive wrist osteomyelitis due to *S. apiospermum* are exceedingly uncommon, making the present case unique in terms of location and etiology. Bone and joint infections caused by molds are often characterized by an indolent clinical course with subtle symptoms, often resulting in delayed diagnosis. Immunosuppression, e.g., after solid organ transplantation, represents the main risk factor for *S. apiospermum* infections. Additional predisposing factors include soil-contaminated wounds and open fractures [4,10]. In this context, the patient was receiving immunosuppressive therapy, which is known to increase susceptibility to opportunistic infections, including invasive fungal infections. In particular, prednisone, belatacept, and mycophenolic acid impair host immune defenses through combined effects on innate immunity and T-cell–mediated responses, thereby facilitating fungal invasion and persistence [14].

In the present case, the chronic course and subtle initial clinical presentation led to a presumed diagnosis of rheumatoid arthritis of the wrist, supported by multiple negative synovial fluid cultures. However, as fungal infection was not suspected, cultures were not specifically plated on Sabouraud agar for fungal culture, limiting their sensitivity. Moreover, failure to challenge the non-infectious differential diagnosis with open bone biopsies led to a six-month delay in initiating appropriate treatment. Because of this diagnostic delay, extensive involvement and destruction of carpal bones were already present at the time of definitive diagnosis, significantly limiting the available therapeutic options.

The diagnosis of proven mold osteomyelitis is based on three pillars. First, imaging plays an essential role in raising suspicion of osteomyelitis, although it cannot establish an etiological diagnosis [15]. Conventional X-ray is only helpful in advanced stages, when bone destruction is already evident. Advanced imaging (i.e., MRI) should be performed as it allows early detection of inflammatory changes and assessment of disease extent, whereas computed tomography better detects bony lesions [6,16]. Second, bone biopsies are key for histopathological examination and microbiological culture on appropriate culture media. Histology may identify acute and chronic inflammation with granulomatous inflammation, and the presence of the typical fungal hyphae within the tissue [15]. The hyphae of *Scedosporium* spp. are septate and branched at 45° angles, similar to aspergillosis, although branching off to the side at 60–70° angle may also be observed. Third, culture is required for genus and species identification and antifungal susceptibility testing. *Scedosporium* spp. grow well on routine fungal media such as Sabouraud agar [15]. Species identification relies on macroscopic and microscopic examination of the colonies, and fungal PCR can be helpful for species identification, particularly when the cultures are negative [6,16].

The management of chronic advanced fungal osteomyelitis in immunocompromised patients represents a major therapeutic challenge.

Treatment typically requires a combination of systemic antifungal treatment and surgical debridement. Voriconazole represents the first-line antifungal treatment for *S. apiospermum* infections and may be combined with liposomal amphotericin B or terbinafine [9]. In immunosuppressed patients treated with calcineurin inhibitors (CNIs), particular attention is required, as voriconazole may significantly increase CNI plasma levels, thus requiring close therapeutic drug monitoring, and when appropriate, adjustment of the CNI dose. In the present case, the patient was on an immunosuppressive regimen without CNIs, therefore clinically relevant drug interactions with voriconazole were not a concern. Treatment duration varies from patient to patient and often extends over several weeks to months, depending on the patient’s immune status and response to therapy [17,18]. Systemic antifungal treatment can be complemented by high-dose local antifungal therapy, as in our patient, in whom a voriconazole-loaded cement spacer was temporarily implanted.

Beyond the direct infectious component, osteomyelitis represents a complex inflammatory process involving the bone and surrounding tissues. The host immune response to infection leads to the release of pro-inflammatory cytokines and activation of immune pathways that, while aimed at pathogen clearance, also contribute to bone destruction and impaired healing [19]. In chronic infections, this inflammatory response may become dysregulated, promoting osteoclast activation, inhibition of osteoblast function, and progressive osteolysis. In addition, persistent infection can induce immune exhaustion and regulatory immune responses, further contributing to chronicity and treatment resistance. These mechanisms are increasingly recognized as key contributors to disease progression and may partly explain the aggressive bone destruction observed in advanced cases [20].

Extensive tissue resection is often necessary to achieve local disease control. However, this may result in considerable functional loss, drastically affecting the patient’s quality of life. In this setting, the decision between limb-salvage and amputation must be carefully individualized. In our patient, this decision was particularly complex due to significant vascular comorbidities and the patient’s repeated refusal of amputation. Although limb salvage was pursued in line with the patient’s preference, the postoperative course was complicated, ultimately leading to flap failure and forearm amputation.

Upper-limb amputation significantly reduces tactile capacity, leading the amputee to rely predominantly on visual feedback. Functional outcomes following amputation are highly variable and depend on several factors, including residual limb function, prosthetic potential, rehabilitation and patient-specific goals. Although preservation of a sensate grasping limb through reconstruction—even with limited function—may offer advantages in selected cases, this must be balanced against the potential benefits of amputation, including a more predictable rehabilitation and earlier functional recovery [21]. In this case, transradial forearm amputation was initially recommended as it would have provided infection control and faster rehabilitation, albeit at the cost of permanent functional loss.

This case highlights the relevance of suspicion for rare fungal pathogens in immunocompromised patients with chronic wrist disease. Given the increased susceptibility and complexity of management in immunosuppressed hosts, early recognition and treatment are key determinants of prognosis and treatment outcomes. Multidisciplinary management is essential to balance infection control, maintenance of transplant immunotolerance, and the implementation of tailored surgical strategies.

## 4. Conclusions

Destructive wrist mold osteomyelitis in immunocompromised hosts represents a challenging infection, with potentially devastating outcomes. Although mold eradication is achievable through a combined surgical and antimicrobial treatment approach, multidisciplinary management is key for a successful outcome. In advanced wrist fungal osteomyelitis with extensive bony tissue loss, the choice between limb salvage and amputation should be individualized, balancing surgical risk, infection control, patient comorbidities, and patient goals. This case underscores the importance of careful patient selection and shared decision-making in complex reconstructive scenarios.

## Figures and Tables

**Table 1:** Antimicrobial susceptibility testing for *Scedosporium apiospermum*.

Antimycotics	MIC
Amphotericin B	32.0 mg/L
Posaconazole	0.25 mg/L
Isavuconazole	0.125 mg/L
Voriconazole	0.032 mg/L

**Table 2:** Antimicrobial susceptibility testing for *Aeromonas veronii*, *Aeromonas hydrophila* and *Morganella morganii*.

Antibiotics	*Aeromonas veronii*	*Aeromonas hydrophila*	*Morganella morganii*
Ampicillin	resistant	resistant	resistant
Amoxicillin–clavulanic acid	susceptible	resistant	resistant
Aztreonam	susceptible	susceptible	-
Cefepime	susceptible	susceptible	susceptible
Ceftazidime	susceptible	resistant	susceptible
Ceftriaxone	susceptible	resistant	susceptible
Ciprofloxacin	resistant	resistant	susceptible
Ertapenem	-	-	susceptible
Gentamicin	susceptible	susceptible	susceptible
Imipenem	susceptible	susceptible	susceptible
Meropenem	susceptible	susceptible	susceptible
Piperacillin–tazobactam	susceptible	susceptible	susceptible
Trimethoprim–sulfamethoxazole	susceptible	susceptible	susceptible

## Data Availability

All relevant data are included in the article. Further inquiries can be directed to the corresponding author.

## Abbreviations

The following abbreviations are used in this manuscript:

MRI	Magnetic resonance imaging
ITS	Internal transcribed spacer
CLSI	Clinical and Laboratory Standards Institute
MIC	Minimum inhibitory concentration
EUCAST	European Committee on Antimicrobial Susceptibility Testing
SD	Surgical debridement
PRC	Proximal row carpectomy
CNIs	Calcineurin inhibitors
PCR	Polymerase chain reaction
